# Liver X Receptor Agonist 4β‐Hydroxycholesterol as a Prognostic Factor in Coronary Artery Disease

**DOI:** 10.1161/JAHA.123.031824

**Published:** 2024-02-23

**Authors:** Roosa Rahunen, Mikko Tulppo, Valtteri Rinne, Samuli Lepojärvi, Juha S. Perkiömäki, Heikki V. Huikuri, Olavi Ukkola, Juhani Junttila, Janne Hukkanen

**Affiliations:** ^1^ Research Unit of Biomedicine and Internal Medicine University of Oulu Oulu Finland; ^2^ Biocenter Oulu University of Oulu Oulu Finland; ^3^ Medical Research Center Oulu Oulu University Hospital and University of Oulu Oulu Finland; ^4^ Admescope (Symeres Finland Ltd) Oulu Finland

**Keywords:** 4β‐hydroxycholesterol, coronary artery disease, liver X receptor, death, sudden cardiac death, Hypertension, Biomarkers, Sudden Cardiac Death

## Abstract

**Background:**

Regardless of progress in treatment of coronary artery disease (CAD), there is still a significant residual risk of death in patients with CAD, highlighting the need for additional risk stratification markers. Our previous study provided evidence for a novel blood pressure–regulating mechanism involving 4β‐hydroxycholesterol (4βHC), an agonist for liver X receptors, as a hypotensive factor. The aim was to determine the role of 4βHC as a prognostic factor in CAD.

**Methods and Results:**

The ARTEMIS (Innovation to Reduce Cardiovascular Complications of Diabetes at the Intersection) cohort consists of 1946 patients with CAD. Men and women were analyzed separately in quartiles according to plasma 4βHC. Basic characteristics, medications, ECG, and echocardiography parameters as well as mortality rate were analyzed. At baseline, subjects with a beneficial cardiovascular profile, as assessed with traditional markers such as body mass index, exercise capacity, prevalence of diabetes, and use of antihypertensives, had the highest plasma 4βHC concentrations. However, in men, high plasma 4βHC was associated with all‐cause death, cardiac death, and especially sudden cardiac death (SCD) in a median follow‐up of 8.8 years. Univariate and comprehensively adjusted hazard ratios for SCD in the highest quartile were 3.76 (95% CI, 1.6–8.7; *P*=0.002) and 4.18 (95% CI, 1.5–11.4; *P*=0.005), respectively. In contrast, the association of cardiac death and SCD in women showed the lowest risk in the highest 4βHC quartile.

**Conclusions:**

High plasma 4βHC concentration was associated with death and especially SCD in men, while an inverse association was detected in women. Our results suggest 4βHC as a novel sex‐specific risk marker of cardiac death and especially SCD in chronic CAD.

**Registration Information:**

clinicaltrials.gov. Identifier NCT01426685.

**Clinical Perspective**

**What Is New?**

- High plasma 4β‐hydroxycholesterol (4βHC) concentration was associated with all‐cause death, cardiac death, and especially sudden cardiac death in male patients with coronary artery disease.
- However, subjects with a high plasma 4βHC concentration had a better cardiovascular risk profile at baseline than subjects in the lower quartiles of plasma 4βHC.
- Plasma 4βHC levels had an inverse association with cardiac death and sudden cardiac death in women, suggesting that 4βHC, an agonist for liver X receptors, could be a novel sex‐specific marker for death, cardiac death, and especially sudden cardiac death in men.

**What Are the Clinical Implications?**

- These results indicate the need to evaluate plasma 4βHC in future studies as a stratification marker for residual risk in male patients with chronic CAD.
- As liver X receptor activation is known to stimulate reverse cholesterol transport but also cardiac lipid accumulation, these findings have implications for liver X receptor as a suggested target in drug development to prevent atherosclerosis and ischemic cardiac events.

**Non standard Abbreviations and Acronyms**
4βHC: 4β‐hydroxycholesterol
4αHC: 4α‐hydroxycholesterol
ARTEMIS: Innovation to Reduce Cardiovascular Complications of Diabetes at the Intersection
LXR: liver X receptor
NCD: noncardiac death
PXR: pregnane X receptor
SCD: sudden cardiac death

Coronary artery disease (CAD) is the leading cause of death globally.^1^ Approximately 18 million people per year die due to cardiovascular diseases, which account an estimated 32% of all deaths worldwide.^2^ Although many of the traditional risk factors for CAD such as smoking, diabetes, hyperlipidemia, hypertension, obesity, and psychological stress are modifiable,^1^ the prevalence of CAD is still remarkably high.^1^ Furthermore, regardless of the great progress in the treatment of CAD and its risk factors, there is still a significant residual risk of cardiac events and death in patients with CAD, suggesting a need for additional risk stratification markers and treatment options.^3^

An oxysterol (oxidation product of cholesterol) 4β‐hydroxycholesterol (4βHC) is formed by cytochrome P450 3A4 and 3A5 enzymes in the liver under the control of nuclear receptors such as pregnane X receptor (PXR; *NR1I2*).^4^, ^5^ Like many other oxysterols, 4βHC is an agonist for liver X receptors (LXRs) α (*NRH13*) and β (*NRH12*).^6^, ^7^ LXRα is expressed in liver, intestine, kidneys, adipose tissue, adrenals, and macrophages, while LXRβ is expressed ubiquitously.^8^ The activation of LXR leads to the upregulation of hepatic lipogenesis, whereas the induction of cholesterol efflux transporters, such as ATP‐binding cassette A1, by LXR activation in peripheral tissues may reduce atherosclerosis via enhanced reverse cholesterol transport.^8^ Incubation with 4βHC induces the efflux of cholesterol and the expression of ATP‐binding cassette A1 and ATP‐binding cassette G1, well‐known LXR targets, in human primary monocyte–derived macrophages and foam cells.^9^ However, 4βHC triggers de novo lipogenesis in mouse hepatocytes in vitro and hepatosteatosis in vivo via both LXRα and LXRβ.^10^ Thus, as an agonist for LXR, 4βHC may have beneficial effects on reverse cholesterol transport and harmful prolipogenic consequences in the liver.

Our previously conducted trial on healthy volunteers demonstrated, unexpectedly, that plasma 4βHC was inversely correlated (*r*=−0.70) with 24‐hour systolic blood pressure, also when plasma 4βHC was elevated >3‐fold by pharmacological activation of PXR.^11^ We have recently explored this finding further by showing that the elevated circulating 4βHC lowers systolic blood pressure in rats, and the higher 4βHC is an independent predictor of lower systolic blood pressure in a cohort of healthy volunteers and patients with obesity, suggesting that 4βHC is a hypotensive factor.^12^ LXR has been previously implicated in the regulation of blood pressure.^13^ As plasma 4βHC levels are repressed by overweight and obesity,^12^, ^14^ we have proposed that 4βHC‐LXR is a novel blood pressure–regulating pathway involved in obesity‐induced hypertension.^12^

This study aims to evaluate the significance of plasma 4βHC as a prognostic factor for cardiac events in a cohort of CAD patients. Furthermore, as 4βHC is an agonist for LXR, but its isomer 4α‐hydroxycholesterol (4αHC) is not, we use plasma 4βHC to interrogate the role of LXR in cardiac events with plasma 4αHC as a negative control.

## Methods

### Study Design and Study Population

The data that support the findings of this study are available from the corresponding author upon reasonable request. The study population comprised of 1946 patients from the ARTEMIS (Innovation to Reduce Cardiovascular Complications of Diabetes at the Intersection; ClinicalTrials.gov identifier: NCT01426685) cohort collected in the Division of Cardiology of Oulu University Hospital (Oulu, Finland). The ARTEMIS study aims to assess several traditional and novel cardiovascular risk markers as determinants of the risk for sudden cardiac death (SCD) in patients with stable CAD. The patients were recruited from 2007 to 2012 from a consecutive series of individuals who had undergone coronary angiography 3 to 6 months earlier. Significant CAD was confirmed by coronary angiography (stenosis >50%). More detailed information on revascularization and enrollment visits are described in the previous ARTEMIS publications.^15^, ^16^, ^17^

Patients aged <18 years or >85 years, New York Heart Association class IV, implantable cardioverter‐defibrillator, and subjects who met the guideline criteria for prophylactic implantation of implantable cardioverter‐defibrillator or end‐stage renal failure needing dialysis were excluded, as well as patients who had a life expectancy <1 year or who were psychologically or physically (due to any other illness) unfit for participation in the study. The study was performed according to the Declaration of Helsinki, and the local committee of research ethics of the Northern Ostrobothnia Hospital District approved the protocol. All subjects provided written informed consent.^16^, ^17^

Since this study concentrates on 4βHC levels and its associations for all‐cause death and cardiac death, there are few additional exclusion criteria. The following 3 features were verified from every subject, and if any were present, the subject was excluded from this study. First, certain traditional epilepsy medications including phenytoin, carbamazepine, oxcarbazepine, eslicarbazepine, phenobarbital, and primidone increase plasma 4βHC levels since these agents act as PXR activators.^18^ Therefore, 15 subjects using these medications were excluded. Epilepsy diagnosis and the use of epilepsy drugs without enzyme‐inducing properties were not an exclusion criterion. Second, since both 4βHC and its isomer 4αHC are formed from cholesterol, 24 subjects who had unusually high levels of plasma 4αHC (which we determined as >15 ng/mL because the mean±SD value in the ARTEMIS cohort was 5.2±2.7 ng/mL) were excluded, because high levels of 4αHC are considered as an indicator for cholesterol degradation due to a poorly preserved sample.^4^ Third, 167 subjects who did not use cholesterol‐lowering drugs were excluded for 2 reasons: high cholesterol level increases 4βHC concentration as it is a formation product of cholesterol, and since this data set comprises only CAD subjects, all individuals would have had an indication for the use of cholesterol‐lowering drugs. Thus, the subjects without cholesterol‐lowering drugs at the baseline could possibly have poor adherence also to other prognostically important drugs diminishing the prognostic value of the baseline plasma 4βHC concentration and therefore skew the outcome results. Cholesterol‐lowering drugs included were mostly statins, but, in addition, a small number of subjects had also ezetimibe (6%), and a few had fibrate or niacin medications. A total of 20 subjects had fibrate, niacin, or ezetimibe without any statin. In total, 206 subjects were excluded, and study population of this study consists of 1740 subjects from the original ARTEMIS data (Figure S1). Since there were two 4βHC samples and three 4αHC samples missing, the number of subjects in analyses were n=1738 and n=1737 for 4βHC and 4αHC, respectively.

### End Points

Our goal was to explore 4βHC as a prognostic factor; therefore, the primary end points were overall deaths, cardiac deaths divided into SCD and non‐SCD, and noncardiac deaths. In addition, resuscitations from cardiac arrest were registered and combined with deaths, SCDs, and cardiac deaths in the statistical analyses, with the aborted SCD being the primary event if the patient eventually died during the follow‐up. The follow‐up information was collected from the national death registries, from the patients by mailed inquiry, from telephone calls to the closest relatives of the deceased victims, and from the electronic patient records. SCD was defined as an unexpected, witnessed death occurring within 1 hour after the onset of symptoms or an unwitnessed death within 24 hours after the patient was last seen alive. The cause of death was defined by an end point committee (J.J., H.V.H.) on the basis of the death certificates, interviews with the closest relatives of the victims, and the autopsy reports. A medicolegal autopsy is mandatory in Finland according to the law, and thus autopsy data were available in most cases.^16^

### Laboratory Assays and Other Clinical Measurements

Laboratory samples were obtained after 12‐hour overnight fast using standardized methods, as described,^19^ and in addition to previously published results, alanine transaminase, γ‐glutamyl transferase and creatinine clearance were measured. Ultra‐high‐performance liquid chromatography coupled with high resolution mass spectrometry was used as previously described^20^ for the measurement of plasma 4αHC and 4βHC at Admescope (Symeres Finland Ltd, Oulu, Finland). Physical exercise capacity was determined as metabolic equivalents (METs), which were calculated from the mean workload during the last minute of a maximal exercise test performed on a stationary bicycle ergometer. After categorization, <5.0 METs was defined as poor, 5.0–6.7 METs as moderate, and >6.7 METs as good exercise capacity tertiles.^15^

### Statistical Analysis

The subjects were divided into quartiles (quartile 1 to quartile 4) according to plasma 4βHC levels (quartile 1 as the lowest, quartile 4 as the highest). All analyses were performed separately for men and women due to a significant sex x 4βHC interaction (*P*<0.01). Sex difference in plasma 4βHC was studied by Mann–Whitney *U* test. Due to the marked sex differences in 4βHC, the subjects were divided to quartiles separately for men and women (quartile 1 to quartile 4) according to plasma 4βHC levels. Thereafter, the sex differences in 4βHC as a predictor for all‐cause death, SCD, non‐SCD, and noncardiac death were evaluated by Cox regression including sex (female sex as a reference category), 4βHC (quartile 1 as a reference category) and sex×4βHC interaction with hazard ratio (HR) and 95% CI.

The total number of subjects was 1192 men and 546 women. The between‐group differences were assessed by 1‐way analysis of variance, Kruskal–Wallis, or χ^2^ test. Between‐group differences (quartiles) were analyzed for the population characteristics, fundamental ECG and echocardiographic parameters, and arrhythmias requiring hospitalization during follow‐up as well as incident type 2 diabetes cases. The end point events all‐cause death, cardiac death (including SCD and non‐SCD), and noncardiac death were analyzed in quartiles. The analyses were repeated with 4αHC quartiles as plasma 4αHC was used as a negative control for the LXR effects of 4βHC.

Univariate Cox regression analysis was performed for SCD. Cox regression was performed, in which age, body mass index, type 2 diabetes, Canadian Cardiovascular Society grading of angina pectoris, left ventricular (LV) ejection fraction, low‐density lipoprotein cholesterol, albumin–creatinine ratio, creatinine clearance, glycated hemoglobin, high‐sensitivity C‐reactive protein, high‐sensitivity troponin, soluble ST2 (suppression of tumorigenicity 2), B‐type natriuretic peptide, and leisure time physical activity were entered in the model as continuous variables when applicable (model 1). The covariates were selected on the basis of the clinical significance and relevant differences found in the characteristics between the groups with and without SCD published recently.^15^, ^16^, ^19^ A second multivariate analysis was performed as model 1 + use of antihypertensive medications (model 2) (β‐blockade, angiotensin‐converting enzyme, angiotensin II receptor, calcium channel blockade, and diuretics). A third multivariate analysis was performed as model 2 + corrected QT interval (QTc) and the presence of T‐wave inversion in inferior limb leads (II, III, aVF) (model 3). The reference categories for men and women were selected as the quartile with the lowest proportion of SCD cases in each sex. Kaplan–Meier analysis was used to illustrate survival curves of the different 4βHC quartiles. In addition, we provide 2 additional analyses as sensitivity analyses, 1 with low‐density lipoprotein cholesterol replaced with total cholesterol (Table S1) and 1 without cholesterol at all as a covariate (Table S2). Furthermore, a spline analysis was performed for SCD for men and women separately, where a spline curve illustrates the risk for SCD as a result of increasing 4βHC levels as a continuous function. The spline analyses and figures were carried out using R version 4.3.0 (R Foundation for Statistical Computing, Vienna, Austria).

The data were analyzed using SPSS software (SPSS Statistics 21, IBM Corp., New York, NY). *P*<0.05 was considered statistically significant.

## Results

### Plasma 4βHC and 4αHC Concentrations and the Cardiovascular Risk Profile

Tables 1 and 2 present the characteristics of the subjects separately in men and women in 4βHC quartiles. In the higher quartiles, quartile 4 and partly in quartile 3, there were healthier subjects in general than in the lower quartiles. Subjects in quartiles 3 and 4 had the lowest body mass index (BMI) values, best physical exercise capacity (MET), lowest prevalence of type 2 diabetes and less use of antihypertensives compared with quartiles 1 and 2. These findings apply for both men and women. However, the lowest total cholesterol and low‐density lipoprotein cholesterol levels were in the lower plasma 4βHC quartiles, quartiles 1 and 2, reflecting the fact that 4βHC is formed from cholesterol. Women had higher 4βHC levels than men (mean, 22% higher), which is in line with the general population.^4^ The median and interquartile range values of 4βHC for men and women were 8.02 (5.90−10.50) and 9.21 (6.67−12.80) (*P*<0.001), respectively. Tables S3 and S4 show results analyzed for plasma 4αHC, used as a negative control. The most significant difference between 4βHC and 4αHC analyses is in the use of antihypertensives; in Tables 1 and 2 (4βHC), the subjects with the most antihypertensive medications are accumulated in the quartiles with lower 4βHC concentrations, while the same trend is not detected in 4αHC analysis (Tables S3 and S4). In 4αHC analysis (Tables S3 and S4), also most of the other previously mentioned variables of interest cluster in the opposite direction to 4βHC analysis (Tables 1 and 2) as both men and women in the higher 4αHC quartiles, quartiles 4 and 3, had the highest BMI values, MET values were worse, and the prevalence of type 2 diabetes was higher compared with the lower quartiles, quartiles 1 and 2. For the assessment between quartiles, Table S5 presents baseline information for 4βHC and 4αHC including the median, minimum to maximum, and interquartile range as well as correlation coefficients between 4βHC, 4αHC, and total cholesterol.

**Table 1:** Characteristics of Patients According to Plasma 4βHC Quartiles at Baseline in Men

Characteristic	Quartile 1, n=299	Quartile 2, n=295	Quartile 3, n=298	Quartile 4, n=300	All men, n=1192
4βHC, minimum–maximum	1.23–5.90	5.91–8.00	8.02–10.4	10.5–35.7	*P* value
Age, y	66±9	66±8	66±9	66±9	0.904
Body mass index, kg/m^2^	29.6±4.6	28.7±4.2	27.6±3.9	26.9±3.9	<0.001
Resting systolic blood pressure, mm Hg	142±22	147±22	143±22	143±23	0.055
Resting diastolic blood pressure, mm Hg	80±12	82±11	80±12	81±11	0.504
Smoker, n (%)	25 (8)	22 (7)	28 (9)	35 (12)	0.319
Alcohol consumers, n (%)	119 (40)	135 (46)	137 (46)	126 (42)	0.337
Servings/wk (if user)	4 (2–9)	5 (3–10)	4 (2–8)	5 (2–10)	0.521
History of AMI, n (%)	158 (53)	145 (49)	154 (52)	153 (51)	0.838
History of PCI/CABG, n (%)	247 (83)	250 (85)	245 (83)	245 (82)	0.794
Syntax score (missing 116)	2 (0–7)	0 (0–5)	1 (0–5)	2 (0–6)	0.023
CCS class ≥2, n (%)	112 (38)	101 (34)	105 (35)	110 (37)	0.849
Leisure time physical activity, n (%)					
Highly active	50 (17)	49 (17)	53 (18)	61 (20)	0.926
Active	111 (37)	112 (38)	105 (35)	105 (35)	
Irregularly active	106 (36)	102 (35)	114 (38)	107 (36)	
Inactive	32 (11)	32 (11)	26 (9)	27 (9)	
Relative METs, n (%)	78(20)	82 (20)	85 (21)	85 (21)	<0.001
Type 2 diabetes, n (%)	170 (57)	125 (42)	114 (38)	102 (34)	<0.001
Duration of diabetes, y	5 (4–12)	5 (1–12)	6 (1–14)	5 (1–12)	0.412
Echocardiogram parameters					
Left ventricular ejection fraction, %	63±10	64±10	63±10	62±10	0.289
Left ventricular mass, g	224±53	224±63	219±56	224±61	0.542
Septal thickness at diastole, mm	11.4±1.9	11.7±2.1	11.3±1.9	11.5±2.0	0.093
Lateral wall thickness, mm	10.6±1.6	10.6±1.6	10.7±1.7	10.7±1.7	0.910
Diastolic function E/E´	10.1±3.2	10.1±3.4	10.0±4.0	9.6±3.5	0.178
Laboratory analyses					
Glycated hemoglobin, mmol/mol	6.6±1.1	6.4±1.1	6.3±1.1	6.1±1.0	<0.001
Total cholesterol, mmol/L	3.4±0.6	3.6±0.6	3.8±0.7	4.2±0.8	<0.001
High‐density lipoprotein, mmol/L	1.1±0.2	1.1±0.3	1.2±0.3	1.3±0.3	<0.001
Low‐density lipoprotein, mmol/L	1.9±0.5	2.0±0.5	2.2±0.5	2.5±0.8	<0.001
Triglycerides, mmol/L	1.2 (0.9–1.8)	1.2 (0.9–1.6)	1.1 (0.9–1.5)	1.1 (0.8–1.6)	0.042
Creatinine clearance, mL/min	106±40	100±33	97±37	93±30	<0.001
U‐albumin/creatinine‐ratio	0.8 (0.6–1.4)	0.8 (0.5–1.1)	0.8 (0.5–1.2)	0.7 (0.5–1.0)	0.012
hs‐CRP, mg/mL	0.9 (0.5–1.9)	0.9 (0.5–1.9)	0.9 (0.4–1.7)	0.9 (0.5–2.2)	0.397
hs‐TnT, ng/L	9 (6–14)	9 (6–14)	9 (6–15)	9 (6–15)	0.857
BNP, ng/L	44 (22–85)	41 (21–89)	43 (24–87)	47 (23–96)	0.659
sST2, ng/L	18 (14–23)	17 (13–23)	17 (14–23)	17 (13–23)	0.278
Gelectin‐3, ng/L	11 (9–13	11 (8–13)	11 (9–13)	11 (9–13)	0.171
ALT, IU/L	29 (22–39)	29 (21–37)	27 (20–37)	26 (20–33)	<0.001
GGT, IU/L	31 (21–48)	30 (20–48)	28 (20–45)	28 (18–43)	0.349
Medication, n (%)					
β‐blockers	282 (94)	257 (87)	261 (88)	257 (86)	0.007
ACE inhibitors or ATII blockers	222 (74)	212 (72)	182 (61)	196 (65)	0.002
Calcium channel blockers	94 (31)	76 (26)	69 (24)	50 (17)	<0.001
Diuretics	113 (38)	91 (31)	79 (27)	84 (28)	0.015
Psychotropic agents	15 (5)	18 (6)	20 (7)	26 (9)	0.333
Electrocardiogram parameters					
Ventricular rate, bpm	60±10	60±10	60±10	60±9	0.613
PQ interval, ms	187±35	185±30	182±30	183±36	0.390
QRS duration, ms	104±17	105±17	106±18	107±17	0.523
QT interval, ms	424±33	422±32	422±33	430±34	0.006
QTc interval, ms	424±26	420±26	419±29	424±27	0.012
T‐wave inversion in leads, n (%)					
II	21 (7.1)	27 (9.4)	33 (11.3)	50 (17.2)	0.001
III	112 (38.1)	118 (41.0)	144 (49.3)	128 (44.1)	0.041
aVF	42 (14.3)	40 (13.9)	54 (18.5)	57 (19.7)	0.146
T‐wave inversion in all leads, n (%)					
II, III, and aVF	10 (3.4)	12 (4.2)	27 (9.2)	35 (12.1)	<0.001

Values are mean±SD, median (first to third quartile), or n (%; within group), unless otherwise specified. 4βHC indicates 4β‐hydroxycholesterol; ACE, angiotensin‐converting enzyme; ALT, alanine aminotransferase; AMI, acute myocardial infarction; ATII, angiotensin II receptor; BNP, B‐type natriuretic peptide; bpm, beats per minute; CABG, coronary artery bypass grafting; CCS, Canadian Cardiovascular Society; GGT, γ‐glutamyl transferase; hs‐CRP, high‐sensitivity C‐reactive protein; hs‐TnT, high‐sensitivity cardiac troponin; MET, metabolic equivalent; PCI, percutaneous coronary intervention; and sST2, soluble suppression of tumorigenicity 2.

**Table 2:** Characteristics of Patients According to Plasma 4β‐HC Quartiles at Baseline in Women

Characteristic	Quartile 1, n=137	Quartile 2, n=136	Quartile 3, n=136	Quartile 4, n=137	All women, n=546
4βHC, minimum–maximum	1.45–6.67	6.68–9.20	9.21–12.7	12.8–49.1	*P* value
Age, y	69±8	70±9	68±8	70±7	0.283
Body mass index, kg/m^2^	30.1±4.9	29.4±4.8	27.8±4.3	26.5±4.9	<0.001
Resting systolic blood pressure, mm Hg	153±24	154±27	154±27	154±27	0.998
Resting diastolic blood pressure, mm Hg	80±11	80±11	80±12	79±13	0.860
Smoker, n (%)	7 (5)	8 (6)	5 (4)	10 (7)	0.337
Alcohol consumer, n (%)	23 (17)	18 (13)	28 (21)	20 (15)	0.379
Servings/week (if user)	2 (1–4)	3 (2–5)	1 (1–3)	2 (1–3)	0.071
History of AMI, n (%)	60 (44)	67 (49)	58 (43)	54 (39)	0.425
History of PCI/CABG	107 (78)	111 (82)	103 (76)	103 (75)	0.569
Syntax score (missing 29)	0 (0–5)	2 (0–5)	0 (0–3)	0 (0–5)	0.048
CCS class ≥2, n (%)	83 (61)	74 (54)	73 (54)	81 (59)	0.535
Leisure time physical activity, n (%)					
Highly active	23 (17)	15 (11)	9 (7)	10 (7)	0.150
Active	52 (38)	54 (40)	46 (34)	49 (36)	
Irregularly active	45 (33)	50 (37)	63 (46)	60 (44)	
Inactive	17 (12)	17 (13)	18 (13)	18 (13)	
Relative METs, n (%)	81 (19)	87 (19)	88 (20)	92 (24)	<0.001
Type 2 diabetes, n (%)	75 (55)	60 (44)	41 (30)	44 (32)	<0.001
Duration of diabetes, y	6 (2–12)	7 (4–12)	4 (2–8)	6 (2–16)	0.166
Echocardiogram parameters					
Left ventricular ejection fraction, %	66±7	67±8	66±8	66±8	0.529
Left ventricular mass, g	177±47	177±45	170±47	168±45	0.289
Septal thickness at diastole, mm	10.6±2.2	10.6±2.1	10.4±2.1	10.3±1.9	0.562
Lateral wall thickness, mm	9.8±1.6	9.8±1.6	9.6±1.6	9.5±1.4	0.519
Diastolic function E/E'	12.1±4.1	12.9±5.0	11.0±3.8	11.6±3.8	0.002
Laboratory analyses					
Glycated hemoglobin, mmol/mol	6.6±1.0	6.4±0.9	6.1±0.7	6.1±0.9	<0.001
Total cholesterol, mmol/L	3.7±0.6	3.9±0.5	4.2±0.7	4.5±0.7	<0.001
High‐density lipoprotein, mmol/L	1.3±0.3	1.3±0.3	1.4±0.3	1.6±0.4	<0.001
Low‐density lipoprotein, mmol/L	2.0±0.5	2.1±0.5	2.4±0.7	2.4±0.7	<0.001
Triglycerides, mmol/L	1.4 (1.0–1.8)	1.3 (1.0–1.6)	1.2 (0.9–1.8)	1.1 (0.9–1.5)	0.002
Creatinine clearance, mL/min	89±28	85±33	80±26	74±24	<0.001
U‐albumin/creatinine‐ratio	1.1 (0.7–1.7)	1.1 (0.8–1.8)	1.1 (0.8–1.5)	1.2 (0.8–1.7)	0.589
hs‐CRP, mg/mL	1.1 (0.6–1.9)	1.1 (0.6–2.2)	0.9 (0.4–1.9)	0.9 (0.4–2.1)	0.142
hs‐TnT, ng/L	7 (5–11)	8 (5–13)	6 (5–11)	7 (5–11)	0.427
BNP, ng/L	50 (32–83)	65 (36–113)	55 (31–96)	65 (40–111)	0.011
sST2, ng/L	16 (13–22)	15 (11–19)	14 (11–19)	14 (11–20)	0.022
Gelectin‐3, ng/L	13 (11–17)	12 (10–15)	12 (10–15)	12 (10–15)	0.008
ALT, IU/L	23 (18–30)	24 (17–31)	24 (18–32)	21 (17–26)	0.271
GGT, IU/L	24 (16–35)	23 (16–33)	22 (18–34)	21 (14–45)	0.801
Medication, n (%)					
β‐blockers	123 (90)	118 (87)	123 (90)	117 (85)	0.520
ACE inhibitors or ATII blockers	107 (76)	97 (71)	92 (68)	91 (66)	0.310
Calcium channel blockers	39 (28)	43 (31)	29 (21)	29 (21)	0.117
Diuretics	67 (49)	61 (45)	53 (39)	51 (37)	0.182
Psychotropic agents	18 (13)	15 (11)	11 (8)	20 (15)	0.368
Electrocardiogram parameters					
Ventricular rate, bpm	62±8	60±10	60±9	61±9	0.368
PQ interval, ms	174±29	183±32	171±29	173±29	0.009
QRS duration, ms	99±17	101±17	99±14	100±18	0.893
QT interval, ms	424±34	435±36	428±32	429±35	0.056
QTc interval, ms	428±27	433±24	426±22	430±24	0.182
T‐wave inversion in leads, n (%)					
II	14 (10.4)	14 (10.9)	15 (11.1)	19 (14.1)	0.780
III	53 (39.3)	52 (40.6)	48 (35.6)	48 (35.6)	0.769
aVF	23 (17.0)	20 (15.6)	18 (13.3)	21 (15.6)	0.867
T‐wave inversion in all leads, n (%)					
II, III, and aVF	8 (5.8)	10 (7.4)	9 (6.6)	14 (10.2)	0.540

Values are mean±SD, median (first to third quartile), or n (%; within group), unless otherwise specified. 4βHC indicates 4β‐hydroxycholesterol; ACE, angiotensin‐converting enzyme; ALT, alanine aminotransferase; AMI, acute myocardial infarction; ATII, angiotensin II receptor; BNP, B‐type natriuretic peptide; bpm, beats per minute; CABG, coronary artery bypass grafting; CCS, Canadian Cardiovascular Society; GGT, γ‐glutamyl transferase; hs‐CRP, high‐sensitivity C‐reactive protein; hs‐TnT, high‐sensitivity cardiac troponin; MET, metabolic equivalent; PCI, percutaneous coronary intervention; and sST2, soluble suppression of tumorigenicity 2.

### Plasma 4βHC Levels and Death

For men and women, all‐cause death; cardiac deaths, including SCD and non‐SCD; and noncardiac deaths were analyzed in 4βHC quartiles with a median follow‐up of 8.8 years (Table 3). Corresponding analyses with 4αHC quartiles are presented in Table 4.

**Table 3:** Mortality Rate According to Plasma 4βHC Quartiles in Men and Women

Mortality	Quartile 1	Quartile 2	Quartile 3	Quartile 4	All	*P* value
Male sex, n	299	295	298	300	1192	
Death, n (%)	54 (18.1)	46 (15.6)	47 (15.8)	71 (23.7)	218 (18.3)	0.036
Cardiac death, n (%)	29 (9.7)	18 (6.1)	16 (5.4)	36 (12.0)	99 (8.3)	0.010
Sudden cardiac death, n (%)	10 (3.3)	8 (2.7)	7 (2.3)	24 (8.0)	49 (4.1)	<0.001
Non–sudden cardiac death, n (%)	19 (6.4)	10 (3.4)	9 (3.0)	12 (4.0)	50 (4.2)	0.190
Noncardiac death, n (%)	25 (8.4)	28 (9.5)	31 (10.4)	35 (11.7)	119 (10.0)	0.486
Female sex, n	137	136	136	137	546	
Death, n (%)	25 (18.2)	24 (17.6)	12 (8.8)	17 (12.4)	78 (14.3)	0.081
Cardiac death, n (%)	10 (7.3)	15 (11.1)	6 (4.4)	4 (2.9)	35 (6.4)	0.033
Sudden cardiac death, n (%)	5 (3.6)	8 (5.9)	2 (1.5)	1 (0.7)	16 (2.9)	0.045
Non–sudden cardiac death, n (%)	5 (3.6)	7 (5.1)	4 (2.9)	3 (2.2)	19 (3.5)	0.533
Noncardiac death, n (%)	15 (10.9)	9 (6.6)	6 (4.4)	13 (9.5)	43 (7.9)	0.187

The values are presented as number of patients (%) of the group. 4βHC indicates 4β‐hydroxycholesterol.

**Table 4:** Mortality Rate According to Plasma 4αHC Quartiles in Men and Women

Mortality	Quartile 1	Quartile 2	Quartile 3	Quartile 4	All	*P* value
Male sex, n	300	296	301	295	1192	
Death, n (%)	57 (19.0)	51 (17.2)	56 (18.6)	54 (18.3)	218 (18.3)	0.952
Cardiac death, n (%)	25 (8.3)	19 (6.4)	29 (9.6)	26 (8.8)	99 (8.3)	0.536
Sudden cardiac death, n (%)	15 (5.0)	8 (2.7)	13 (4.3)	13 (4.4)	49 (4.1)	0.540
Non–sudden cardiac death, n (%)	10 (3.3)	11 (3.7)	16 (5.3)	13 (4.4)	50 (4.2)	0.667
Noncardiac death, n (%)	32 (10.7)	32 (10.8)	27 (9.0)	28 (9.5)	119 (10.0)	0.895
Female sex, n	137	136	137	135	545	
Death, n (%)	21 (15.3)	18 (13.2)	20 (14.6)	19 (14.1)	78 (14.3)	0.967
Cardiac death, n (%)	10 (7.3)	6 (4.4)	8 (5.8)	11 (8.1)	35 (6.4)	0.607
Sudden cardiac death, n (%)	6 (4.4)	3 (2.2)	2 (1.5)	5 (3.7)	16 (2.9)	0.478
Non–sudden cardiac death, n (%)	4 (2.9)	3 (2.2)	6 (4.4)	6 (4.4)	19 (3.5)	0.705
Noncardiac death, n (%)	11 (8.0)	12 (8.8)	12 (8.8)	8 (5.9)	43 (7.9)	0.832

The values are presented as number of patients (%) of the group. 4αHC indicates 4α‐hydroxycholesterol.

**Figure 1:**
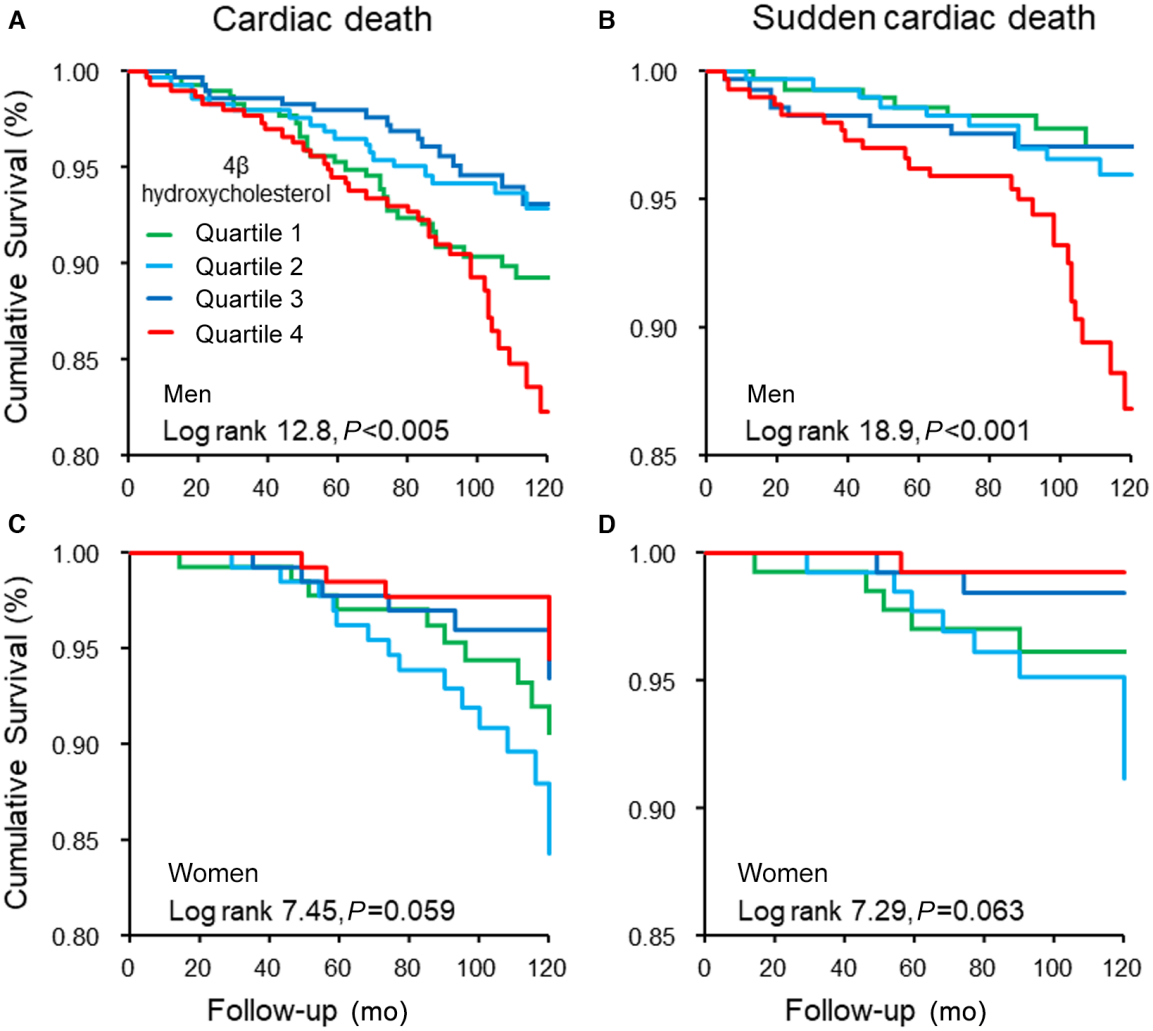
Cardiac deaths and sudden cardiac deaths in men and women according to plasma 4β‐hydroxycholesterol quartiles in 8.8 years of median follow‐up. **A**, Cardiac deaths in men (n=99). **B**, Sudden cardiac deaths in men (n=49). **C**, Cardiac deaths in women (n=35). **D**, Sudden cardiac deaths in women (n=16).

In men, high levels of plasma 4βHC were associated with all‐cause death, cardiac deaths, and SCD, and most deaths and cardiac deaths were in quartile 4. For all‐cause death, 32.6% of deaths were in quartile 4, and 24.8% in quartile 1 (*P*=0.036). For cardiac deaths, 36.4% were in quartile 4, and 29.3% in quartile 1 (*P*=0.010). This trend was more pronounced for SCD, as 49.0% of SCDs were in quartile 4, and only 20.4% of cases were in quartile 1. Figure 1A presents the data of cardiac deaths in the Kaplan–Meier curve in men. In women, the associations of cardiac deaths and SCDs were statistically significant (*P*=0.033 and *P*=0.045, respectively) between the quartiles, but the mortality trend was contrary to that of men. Figure 1C presents the data of cardiac deaths in the Kaplan–Meier curve in women. None of these analyses were statistically significant when analyzed with 4αHC quartiles in men or women. Furthermore, new atrial fibrillation in 2‐year follow‐up, any hospitalization for arrhythmia, incident type 2 diabetes in 5‐year follow‐up, and incident myocardial infarction (MI) in 5‐year follow‐up did not seem to explain the high incidence of SCDs in upper quartiles, and cases with these events accumulated in the lower quartiles.

### Plasma 4βHC and ECG Parameters at Baseline

Several ECG parameters were analyzed at the baseline (Tables 1 and 2) with T‐wave inversions in inferior leads II, III and aVF, and QTc interval demonstrating associations with 4βHC quartiles. In men, most of the inferior T‐wave inversions were present in quartile 3 and 4 groups. T‐wave inversions in the inferior leads II, III and aVF were analyzed separately and in combination (all 3 inferior leads) (Tables 1 and 2). For lead II, 38.2% of all subjects who had T‐wave inversion were in quartile 4, whereas quartile 1 had only 16.0% of the T‐wave inversion cases (*P*<0.001). For lead III, 28.7% of the T‐wave inversion cases were in quartile 3, whereas quartile 1 had 22.3% of the cases (*P*=0.041). For aVF, there were no statistically significant differences between 4βHC quartiles. The subjects with T‐wave inversion in all 3 inferior leads accumulated in upper quartiles; 41.7% of subjects who had T‐wave inversions in all 3 inferior leads were in quartile 4, whereas only 11.9% of these cases were in quartile 1 (*P*<0.0001). In men, QTc interval appeared as a U‐shaped pattern between 4βHC quartiles. In quartile 1, QTc interval was mean, 424 ms; quartile 2 to quartile 3, 420–419 ms; and quartile 4, 424 ms (*P*<0.001). In women, there were no statistically significant differences between 4βHC quartiles in any of the inferior lead T‐wave inversions or QTc interval.

In the control analyses with 4αHC quartiles in men, there were statistically significant differences between the quartiles in T‐wave inversions in lead II, but not in any other lead or the combination of 3 leads (II, III, aVF). In QTc interval, there were statistically significant differences between quartiles, with a rising trend toward quartile 4, from quartile 1 (418 ms) to quartile 4 (426 ms; *P*=0.003).

Several essential echocardiographic parameters were analyzed in 4βHC quartiles: LV ejection fraction (%), LV mass (g), septal thickness at diastole (mm), lateral wall thickness (mm), and diastolic function E/E′; none seemed to explain the high incidence of SCDs in upper quartiles.

### Sex Differences in SCD Risk Related to Plasma 4βHC Levels

Cox regression analysis results in significant sex×4βHC interaction for all‐cause death (HR, 2.08 [95% CI, 1.02–4.23]; *P*=0.043) and for SCD (HR, 12.8 [95% CI, 1.32–123.9]; *P*=0.028) between the lowest and the highest 4βHC quartiles but not with non‐SCD or noncardiac death.

Since significant association between 4βHC levels and SCD was apparent in end point analyses, we conducted survival analyses for SCD. Results from Cox regression analysis for 4βHC quartiles and SCD were performed for both men and women (Table 5; Tables S1 and S2). In men, the optimal cutoff point value was estimated as >11.0 ng/mL and reference quartile as quartile 3. Above the cutoff value, the HR for SCD based on only the 4βHC levels (univariate model) was 3.27 (*P*<0.001), and HR for quartile 4 was 3.76 (*P*=0.002). In model 1 adjusted for multiple variables (see Methods), HR in quartile 4 was 3.45 (*P*=0.009); in the multivariate model 2, with the use of antihypertensive medications added to the model, HR was 3.47 in quartile 4 (*P*=0.009); and in model 3, where T‐wave inversions and QTc interval were added to the model, HR was 4.18 (*P*=0.005). Figure 1B presents the survival function of SCD in the Kaplan–Meier curve in the quartiles in men. In women, there was a statistically significant risk for SCD below the cutoff value (<9.7 ng/mL) in univariate analysis and all models 1 through 3, and in quartile 2 in the models 1 through 3. Thus, the highest SCD risk tended to concentrate on the quartiles with lower 4βHC concentrations, in contrast with men. Figure 1D presents the survival function of SCD in the Kaplan–Meier curve in the quartiles in women.

**Table 5:** Cox Regression Analysis for 4β‐HC and Sudden Cardiac Death

4βHC	Sudden cardiac death							
	Univariate		Model 1		Model 2		Model 3	
	Hazard ratio (95% CI)	*P* value	Hazard ratio (95% CI)	*P* value	Hazard ratio (95% CI)	*P* value	Hazard ratio (95% CI)	*P* value
	Men							
Continuous (ln)	2.84 (1.53–5.26)	<0.001	1.78 (0.88–3.60)	0.107	1.82 (0.91–3.69)	0.092	1.86 (0.91–3.81)	0.089
Quartile 1	1.35 (0.52–3.56)	0.538	1.71 (0.61–4.80)	0.303	1.69 (0.60–4.74)	0.424	1.99 (0.67–5.9)	0.218
Quartile 2	1.13 (0.41–3.12)	0.809	1.57 (0.53–4.61)	0.409	1.55 (0.53–4.55)	0.357	1.68 (0.52–5.4)	0.385
Quartile 3	Reference		Reference		Reference		Reference	
Quartile 4	3.76 (1.62–8.72)	0.002	3.45 (1.35–8.80)	0.009	3.47 (1.35–8.90)	0.009	4.18 (1.52–11.4)	0.005
Cutoff >11.0 ng/mL	3.27 (1.85–5.75)	<0.001	2.62 (1.33–5.08)	0.005	2.63 (1.34–5.16)	0.005	2.96 (1.48–5.90)	0.002
	Women							
Continuous (ln)	0.41 (0.15–1.12)	0.083	0.43 (0.13–1.41)	0.163	0.45 (0.14–1.45)	0.181	0.45 (0.14–1.45)	0.183
Quartile 1	4.51 (0.52–38.7)	0.170	6.74 (0.55–81.8)	0.054	7.83 (0.59–104)	0.119	13.7 (0.59–320)	0.103
Quartile 2	7.79 (0.97–62.4)	0.052	10.1 (0.97–105)	0.048	12.6 (1.09–146)	0.043	25.1 (1.51–545)	0.040
Quartile 3	1.97 (0.17–21.7)	0.579	3.15 (0.25–39.2)	0.372	3.61 (0.28–47.0)	0.326	5.40 (0.28–104)	0.264
Quartile 4	Reference		Reference		Reference		Reference	
Cutoff <9.7 ng/mL	5.57 (1.26–24.5)	0.023	5.64 (1.05–30.3)	0.044	6.81 (1.15–40.4)	0.035	9.73 (1.19–79.4)	0.034

Hazard ratios with 95% CIs were calculated by univariate Cox regression analysis for 4βHC and sudden cardiac death. Cox regression where age, body mass index, type 2 diabetes, Canadian Cardiovascular Society grading of angina pectoris, left ventricular ejection fraction, low‐density lipoprotein cholesterol, albumin‐creatinine ratio, creatinine clearance, glycated hemoglobin, high‐sensitivity C‐reactive protein, high‐sensitivity troponin, soluble ST2, B‐type natriuretic peptide, and leisure time physical activity were entered in the model as continuous variables when applicable (model 1). The second multivariate analysis (model 2) was performed as model 1 + use of antihypertensive medication (beta‐adrenergic blocking agent, angiotensin‐converting enzyme inhibitor, angiotensin II receptor blocker, calcium channel blocker, and diuretics). The third multivariate analysis (model 3) was performed as model 2 + QTc interval and presence of T‐wave inversions in all inferior leads (II, III, and aVF).

**Figure 2:**
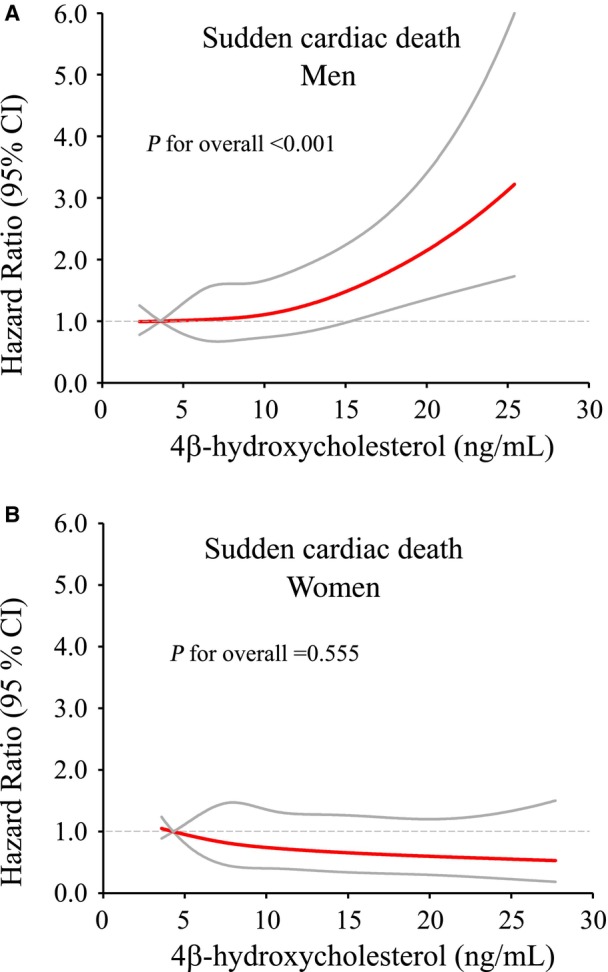
Spline curves for sudden cardiac death depending on 4β‐hydroxycholesterol levels. Red line presents hazard ratio, and gray lines 95% CI. **A**, Spline curve for men. **B**, Spline curve for women.

The curves produced by spline analysis illustrate the risk for SCD as a result of increasing 4βHC levels as a continuous function. Figure 2 presents curves for men (Figure 2A) and women (Figure 2B). Figure 2A shows that the risk for SCD begins to increase after 5 ng/mL and is statistically significant above a 4βHC level of 15 ng/mL, which corresponds to quartile 4. In women, the risk for SCD tends to decrease above 5 ng/mL (Figure 2B), which corresponds to quartile 1.

## Discussion

In the present study with a cohort of patients with CAD with 8.8 years of median follow‐up, high plasma 4βHC levels associated with all‐cause death, cardiac death, and especially SCD in men, even though the patients in the upper plasma 4βHC quartile were healthier than patients in the lower 4βHC quartiles as assessed with the traditional markers such as BMI, METs, glycated hemoglobin, triglycerides, and the prevalence of type 2 diabetes. High plasma 4βHC concentration was associated with better overall health in women. However, the women in the highest plasma 4βHC quartile had the lowest risk of cardiac death and SCD, in contrast with men. Thus, we suggest that plasma 4βHC is a novel sex‐specific predictor of death, cardiac death, and especially SCD in patients with chronic CAD.

It is known that 4βHC is an agonist for LXRα and LXRβ.^6^, ^7^ LXR regulates the beneficial reverse cholesterol transport with promising antiatherogenic effects in animal models.^8^, ^21^ LXRα and LXRβ mRNA and protein are expressed in mice hearts,^22^ and mRNA expression levels of both LXRs are >10‐fold higher in fibroblasts and endothelial cells compared with myocytes.^23^ LXRα mRNA is also expressed in the human heart,^24^ and mRNA and protein of both LXRs are expressed in human endothelium.^25^ LXRα protein is highly expressed in macrophages present in the atherosclerotic lesions of the human aorta, but not in the normal aorta.^26^ Ritter et al^27^ administered LXR agonist AZ876 to male mice, leading to increased expression of LXR target genes responsible for the synthesis of omega‐3 fatty acids in the left ventricle.^27^ Liquid chromatography–high resolution mass spectrometry–based lipidomics demonstrated that mice treated with LXR agonist possessed a higher quantity of monounsaturated fatty acids (especially oleic acid) and polyunsaturated fatty acids (especially the omega‐3 fatty acid docosahexaenoic acid compared with control mice).^27^ Lei et al^23^ incubated murine hearts with LXR agonist GW3965, which induced the expression of mRNA of LXR target genes in cultured cardiomyocytes, and the target gene response was verified in HL‐1 cells, an immortalized mouse cardiomyocyte cell line. GW3965 incubation with HL‐1 cells increased the amount of intracellular lipid droplets; in vivo GW3965 injection induced LXR target genes, and mice dosed with GW3965 had 76% more lipid droplets in the left ventricle compared with control mice.^23^ LXR activation is considered cardioprotective as it protects mice against myocardial ischemia–reperfusion injury.^22^, ^23^ LXR activation has also been proposed to be antihypertrophic, anti‐inflammatory, antiapoptotic, antifibrotic, and proangiogenic.^13^ Although cardiac LXR activation is beneficial during the acute ischemia–reperfusion or catecholamine‐mediated cardiac damage in animal models, our study suggests that LXR activation by elevated 4βHC may expose to increased risk of death and SCD in male patients with chronic CAD, perhaps via cardiac lipid accumulation demonstrated in rodent and cell models.

Regarding 4βHC, we have previously shown that about 9‐fold elevation of serum 4βHC by the PXR activator pregnenolone 16a‐carbonitrile led to induction of mRNA and protein of ATP‐binding cassette A1 and ATP‐binding cassette G1, typical LXR targets, in the rat left ventricle,^9^ suggesting that 4βHC can regulate the expression of LXR targets in the heart. It has also been demonstrated that 4βHC promotes hepatic lipid‐droplet formation and triglyceride accumulation in mice by inducing SREBP1c, the master lipogenic transcription factor, by acting as an agonist for LXRα and LXRβ.^10^ LXR activation is known to induce SREBP1c in mice hearts.^23^ Intriguingly, in a swine study with 8‐week high‐caloric and ‐cholesterol diet, high‐caloric and ‐cholesterol diet + ezetimibe, and control diet groups, 4βHC concentration in the left ventricle was positively correlated with the epicardial fat thickness, the thickness of the interventricular septum and LV posterior wall as well as the relative wall thickness and LV ejection fraction, and negatively correlated with the diastolic function.^28^ Surprisingly, these associations seemed to be 4βHC specific, as no other oxysterol (11 other oxysterols measured) was correlated with these echocardiographic parameters.^28^ In our study, the interventricular septum, LV lateral wall, LV ejection fraction, and diastolic function were measured; in men, there were no statistically significant differences among 4βHC quartiles, whereas in women high plasma 4βHC was associated with better diastolic function.

As mentioned above, in mice, LXR activation increases lipid droplets in the heart and especially omega‐3 fatty acid docosahexaenoic acid content.^27^ Traditional thinking that omega‐3 fatty acids are beneficial for cardiovascular health have been recently challenged, including a meta‐analysis with 5 randomized controlled trials consisting of 50 277 study subjects in total.^29^ Subjects with cardiovascular disease or a high risk for cardiovascular disease randomized to an omega‐3 fatty acid supplement had significantly increased risk for incident atrial fibrillation compared with placebo.^29^ This finding provides evidence that cardiac accumulation of fatty acids may have negative proarrhythmic consequences in the context of CAD. The mechanistic and epidemiological connection with atrial fibrillation and SCD have been explored in multiple studies.^30^, ^31^, ^32^ From a mechanistic point of view, atrial fibrillation can be an underlying condition that creates a susceptible starting substrate for SCD, or it can be a triggering element for ventricular arrhythmias.^33^ Although there was no direct evidence of proarrhythmic effects of elevated 4βHC in our study, SCD is often caused by arrhythmia in the population with CAD.^34^ We are theorizing that LXR activation‐elicited lipid droplet and docosahexaenoic acid accumulation could contribute to the observed association between high plasma 4βHC and SCD in men, with the previously noted sex differences in LXR biology following MI, possibly explaining the sex difference in the direction of the association between plasma 4βHC and SCD (see below).

In the present study, the proportion of the subjects with T‐wave inversions in the inferior leads at the baseline were strongly positively associated with plasma 4βHC in men but not in women. Thus, the male participants deemed the fittest on the basis of traditional markers of physical fitness, had the highest plasma 4βHC, the most inferior T‐wave inversions and the highest risk of SCD.^35^, ^36^ Multiple T‐wave inversions in an isolated region are related to SCD; for example, Haukilahti et al^37^ explored previously recorded ECG findings from autopsy‐verified individuals with SCD in the Fingesture study, which included 5869 individuals with SCD and 7217 control subjects. Isolated region T‐wave inversions (T‐wave inversion was interpreted as isolated if there were at least 2 T‐wave inversions of −0.1 mV or greater in at least 2 contiguous leads) were more common in individuals with SCD compared with controls, as well as when the inversions were in the inferior region. Isolated region T‐wave inversion was also more in common in individuals with ischemic SCD compared with individuals with nonischemic SCD, again also when the inversions were in the inferior region. Further, isolated T‐wave inversion in any lead (region) were more common in men with SCD compared with women.^37^ Furthermore, in a study consisting of 10 889 middle‐aged Finnish subjects from the general population, the prevalence of T‐wave inversions in any lead was low (1.2%), and especially T‐wave inversions in leads other than right precordial leads were risk markers for all‐cause and cardiac death as well as SCD.^35^ However, it should be noted that HR for SCD was not attenuated in our study when T‐wave inversions and QTc interval were added to the model. This may imply that 4βHC has some other SCD‐promoting mechanism than the increased prevalence of T‐wave inversions.

Our recent study^12^ suggested that 4βHC is a hypotensive factor, plasma 4βHC levels are repressed by overweight and obesity, and 4βHC is part of a PXR‐LXR‐4βHC pathway involved in obesity‐induced hypertension. The same impact is seen in this study as BMI is decreasing toward the upper quartiles with higher 4βHC levels, and a strong similar gradient for type 2 diabetes is notable. It is well known that high BMI is a risk factor for type 2 diabetes^38^; therefore, it is not surprising that high BMI and the prevalence of type 2 diabetes cases are accumulated in the lower quartiles. The gradient for BMI and type 2 diabetes is noted for 4βHC levels and not observed for plasma 4αHC. This possibly reflects the difference in the effect of overweight and obesity on their modes of formation (enzymatic versus nonenzymatic).^4^

Finally, the plasma levels of 4αHC, an isomer of 4βHC, did not have an association with all‐cause death, cardiac death, or SCD in men or women. We used 4αHC as a negative control for LXR‐activating effects of 4βHC since 4αHC is not an agonist for LXRs.^7^ Thus, we suggest that our results support the existence of the 4βHC‐LXR pathway in the deleterious association of 4βHC with SCD.

The most intriguing finding in our study is the sex specificity of the association of 4βHC with SCD. It is known that women have higher plasma concentrations of 4βHC,^4^ as also demonstrated here, and pregnancy further elevates plasma 4βHC progressively >2‐fold in the third trimester.^36^ The expression of LXRβ protein is lower in hypothalamus of female rats compared with male rats, while there is no difference between LXRα protein expression.^39^ There are no publications on the sex specificity of LXR expression in human or rodent hearts, to the best of our knowledge. However, a translational study with data from post‐MI young and old mice and human patients following MI revealed that old female mice lost the ability to activate LXR/RXR signaling pathway in the heart compared with young female mice, while old male mice retained the ability to activate LXR/RXR signaling after MI.^40^ In humans, men with MI + heart failure had upregulation, whereas women with MI + heart failure had downregulation of the LXR/RXR pathway, based on plasma glycoproteomics.^40^ Thus, there is evidence for sex specificity in LXR signaling following MI. According to the Fingesture study,^41^ women with SCD were significantly older and had a greater prevalence of primary myocardial fibrosis and nonischemic causes of SCD compared with men. Also, women were more likely to have normal ECG findings and no autopsy findings in the heart.^41^ Since LXR activation is hypothesized to have many systemic effects, including an antifibrotic effect,^13^ there could be a possible connection between female mice and women losing their ability to activate LXR/RXR signaling pathway following MI with advancing age, and women with SCD being older and possessing more primary myocardial fibrosis than men.

There are some limitations in our study. For example, there are more than twice as many men as women in the study population, with the proportion of men being 69%. As SCD is a rare event, the number of SCDs is quite small, with the preponderance of events in men (16 events in women, 49 events in men), although the difference is not that significant percentage‐wise (4.1% in men and 2.9% in women). Furthermore, the study population is relatively small to assess a rare event, and we had no access to a suitable replication cohort.

## Conclusions

This study evaluated plasma 4βHC as a prognostic factor in a cohort of patients with CAD. High levels of plasma 4βHC, an agonist for LXR, were associated with all‐cause death, cardiac death, and SCD in male patients with CAD, although men with the highest plasma 4βHC levels were physically the fittest. In contrast with men, women with the highest 4βHC concentrations were not only the fittest but had the lowest risk of cardiac death and SCD. Thus, we suggest that plasma 4βHC is a novel sex‐specific predictor of death, cardiac death, and especially SCD in patients with chronic CAD. The mechanism of high plasma 4βHC–elicited risk of SCD in men could involve the higher proportion of inferior T‐wave inversions and possibly LXR activation–mediated fatty acid accumulation in the heart. Our findings require more mechanistic research and replication in additional CAD cohorts in future.

## Sources of Funding

The study was supported by the Finnish Foundation for Cardiovascular Research, Finnish Government Grants for Health Research, Urmas Pekkala Foundation, Academy of Finland, Sigrid Juselius Foundation, Finnish Technology Development Centre, the Finnish Medical Foundation, and Gust.Rud.Idman Foundation.

## Disclosures

V. Rinne is employed by Symeres Finland, operating under the brand name Admescope, and is a minor shareholder of Symeres. The remaining authors have no disclosures to report.

## Supporting information

Tables S1–S5
Figure S1

## References

[1] Malakar AK , Choudhury D , Halder B , Paul P , Uddin A , Chakraborty S . A review on coronary artery disease, its risk factors, and therapeutics. J Cell Physiol. 2019;234:16812–16823. doi: 10.1002/jcp.28350 30790284

[2] Cardiovascular diseases. World Health Organization. Accessed October 3, 2022. https://www.who.int/health‐topics/cardiovascular‐diseases#tab=tab_1

[3] Lawler PR , Bhatt DL , Godoy LC , Lüscher TF , Bonow RO , Verma S , Ridker PM . Targeting cardiovascular inflammation: next steps in clinical translation. Eur Heart J. 2021;42:113–131. doi: 10.1093/eurheartj/ehaa099 32176778

[4] Diczfalusy U , Nylén H , Elander P , Bertilsson L . 4β‐Hydroxycholesterol, an endogenous marker of CYP3A4/5 activity in humans. Br J Clin Pharmacol. 2011;71:183–189. doi: 10.1111/j.1365-2125.2010.03773.x 21219398 PMC3040538

[5] Zhou S‐F . Drugs behave as substrates, inhibitors and inducers of human cytochrome P450 3A4. Curr Drug Metab. 2008;9:310–322. doi: 10.2174/138920008784220664 18473749

[6] Janowski BA , Willy PJ , Devi TR , Falck JR , Mangelsdorf DJ . An oxysterol signalling pathway mediated by the nuclear receptor LXR alpha. Nature. 1996;383:728–731. doi: 10.1038/383728a0 8878485

[7] Nury T , Samadi M , Varin A , Lopez T , Zarrouk A , Boumhras M , Riedinger JM , Masson D , Vejux A , Lizard G . Biological activities of the LXRα and β agonist, 4β‐hydroxycholesterol, and of its isomer, 4α‐hydroxycholesterol, on oligodendrocytes: effects on cell growth and viability, oxidative and inflammatory status. Biochimie. 2013;95:518–530. doi: 10.1016/j.biochi.2012.11.013 23220593

[8] Lee SD , Tontonoz P . Liver X receptors at the intersection of lipid metabolism and atherogenesis. Atherosclerosis. 2015;242:29–36. doi: 10.1016/j.atherosclerosis.2015.06.042 26164157 PMC4546914

[9] Salonurmi T , Nabil H , Ronkainen J , Hyötyläinen T , Hautajärvi H , Savolainen MJ , Tolonen A , Orešič M , Känsäkoski P , Rysä J , et al. 4 β‐Hydroxycholesterol signals from the liver to regulate peripheral cholesterol transporters. Front Pharmacol. 2020;11:361. doi: 10.3389/FPHAR.2020.00361 32292343 PMC7118195

[10] Moldavski O , Zushin PJH , Berdan CA , Van Eijkeren RJ , Jiang X , Qian M , Ory DS , Covey DF , Nomura DK , Stahl A , et al. 4β‐Hydroxycholesterol is a prolipogenic factor that promotes SREBP1c expression and activity through the liver X receptor. J Lipid Res. 2021;62:62. doi: 10.1016/J.JLR.2021.100051 PMC8042401 33631213

[11] Hassani‐Nezhad‐Gashti F , Salonurmi T , Hautajärvi H , Rysä J , Hakkola J , Hukkanen J . Pregnane X receptor activator rifampin increases blood pressure and stimulates plasma renin activity. Clin Pharmacol Ther. 2020;108:856–865. doi: 10.1002/cpt.1871 32344455

[12] Rahunen R , Kummu O , Koivukangas V , Hautajärvi H , Hakkola J , Rysä J , Hukkanen J . Pregnane X receptor−4β‐hydroxycholesterol Axis in the regulation of overweight‐and obesity‐induced hypertension. J Am Heart Assoc. 2022;11:e23492. doi: 10.1161/JAHA.121.023492 PMC9075316 35229613

[13] Cannon MV , van Gilst WH , de Boer RA . Emerging role of liver X receptors in cardiac pathophysiology and heart failure. Basic Res Cardiol. 2016;111:1–17. doi: 10.1007/s00395-015-0520-7 26611207 PMC4661180

[14] Eide Kvitne K , Hole K , Krogstad V , Wollmann BM , Wegler C , Johnson LK , Hertel JK , Artursson P , Karlsson C , Andersson S , et al. Correlations between 4β‐hydroxycholesterol and hepatic and intestinal CYP3A4: protein expression, microsomal ex vivo activity, and in vivo activity in patients with a wide body weight range. Eur J Clin Pharmacol. 2022;78:1289–1299. doi: 10.1007/s00228-022-03336-9 35648149 PMC9283167

[15] Tulppo MP , Kiviniemi AM , Lahtinen M , Ukkola OH , Toukola T , Perkiömäki JS , Junttila MJ , Huikuri HV . Physical activity and the risk for sudden cardiac death in patients with coronary artery disease. Circ Arrhythm Electrophysiol. 2020;13:505–513. doi: 10.1161/CIRCEP.119.007908 32433894

[16] Junttila MJ , Kiviniemi AM , Lepojärvi ES , Tulppo MP , Piira OP , Kenttä T , Perkiömäki JS , Ukkola OH , Myerburg RJ , Huikuri HV . Type 2 diabetes and coronary artery disease: preserved ejection fraction and sudden cardiac death. Heart Rhythm. 2018;15:1450–1456. doi: 10.1016/j.hrthm.2018.06.017 30274618

[17] Karjalainen JJ , Kiviniemi AM , Hautala AJ , Piira OP , Lepojärvi ES , Peltola MA , Ukkola OH , Hedberg PSM , Huikuri HV , Tulppo MP . Determinants and prognostic value of cardiovascular autonomic function in coronary artery disease patients with and without type 2 diabetes. Diabetes Care. 2014;37:286–294. doi: 10.2337/dc13-1072 23959565

[18] Hukkanen J . Induction of cytochrome P450 enzymes: a view on human in vivo findings. Expert Rev Clin Pharmacol. 2012;5:569–585. doi: 10.1586/ecp.12.39 23121279

[19] Lepojärvi ES , Huikuri HV , Piira OP , Kiviniemi AM , Miettinen JA , Kenttä T , Ukkola OH , Perkiömäki JS , Tulppo MP , Junttila MJ . Biomarkers as predictors of sudden cardiac death in coronary artery disease patients with preserved left ventricular function (ARTEMIS study). PloS One. 2018;13:13. doi: 10.1371/JOURNAL.PONE.0203363 PMC6143233 30226845

[20] Hautajärvi H , Hukkanen J , Turpeinen M , Mattila S , Tolonen A . Quantitative analysis of 4β‐ and 4α‐hydroxycholesterol in human plasma and serum by UHPLC/ESI‐HR‐MS. J Chromatogr B Analyt Technol Biomed Life Sci. 2018;1100–1101:179–186. doi: 10.1016/j.jchromb.2018.09.028 30340067

[21] Hong C , Tontonoz P . Liver X receptors in lipid metabolism: opportunities for drug discovery. Nat Rev Drug Discov. 2014;13:433–444. doi: 10.1038/nrd4280 24833295

[22] He Q , Pu J , Yuan A , Lau WB , Gao E , Koch WJ , Ma XL , He B . Activation of liver‐X‐receptor α but not liver‐X‐receptor β protects against myocardial ischemia/reperfusion injury. Circ Heart Fail. 2014;7:1032–1041. doi: 10.1161/CIRCHEARTFAILURE.114.001260 25277999 PMC4527689

[23] Lei P , Baysa A , Nebb HI , Valen G , Skomedal T , Osnes JB , Yang Z , Haugen F . Activation of liver X receptors in the heart leads to accumulation of intracellular lipids and attenuation of ischemia‐reperfusion injury. Basic Res Cardiol. 2013;108:13. doi: 10.1007/S00395-012-0323-Z 23266787

[24] Chen M , Beaven S , Tontonoz P . Identification and characterization of two alternatively spliced transcript variants of human liver X receptor alpha. J Lipid Res. 2005;46:2570–2579. doi: 10.1194/jlr.M500157-JLR200 16170053

[25] Morello F , Saglio E , Noghero A , Schiavone D , Williams TA , Verhovez A , Bussolino F , Veglio F , Mulatero P . LXR‐activating oxysterols induce the expression of inflammatory markers in endothelial cells through LXR‐independent mechanisms. Atherosclerosis. 2009;207:38–44. doi: 10.1016/j.atherosclerosis.2009.04.001 19426978

[26] Watanabe Y , Jiang S , Takabe W , Ohashi R , Tanaka T , Uchiyama Y , Katsumi K , Iwanari H , Noguchi N , Naito M , et al. Expression of the LXRalpha protein in human atherosclerotic lesions. Arterioscler Thromb Vasc Biol. 2005;25:622–627. doi: 10.1161/01.ATV.0000154489.53077.4e 15625283

[27] Ritter D , Goeritzer M , Thiele A , Blumrich A , Beyhoff N , Luettges K , Smeir E , Kasch J , Grune J , Müller OJ , et al. Liver X receptor agonist AZ876 induces beneficial endogenous cardiac lipid reprogramming and protects against isoproterenol‐induced cardiac damage. J Am Heart Assoc. 2021;10:e019473. doi: 10.1161/JAHA.120.019473 34227403 PMC8483473

[28] Shimabukuro M , Okawa C , Yamada H , Yanagi S , Uematsu E , Sugasawa N , Kurobe H , Hirata Y , Kim‐Kaneyama J , Lei XF , et al. The pathophysiological role of oxidized cholesterols in epicardial fat accumulation and cardiac dysfunction: a study in swine fed a high caloric diet with an inhibitor of intestinal cholesterol absorption, ezetimibe. J Nutr Biochem. 2016;35:66–73. doi: 10.1016/j.jnutbio.2016.05.010 27416363

[29] Lombardi M , Carbone S , Del Buono MG , Chiabrando JG , Vescovo GM , Camilli M , Montone RA , Vergallo R , Abbate A , Biondi‐Zoccai G , et al. Omega‐3 fatty acids supplementation and risk of atrial fibrillation: an updated meta‐analysis of randomized controlled trials. Eur Heart J Cardiovasc Pharmacother. 2021;7:e69–e70. doi: 10.1093/ehjcvp/pvab008 33910233 PMC8302253

[30] Marijon E , Le Heuzey JY , Connolly S , Yang S , Pogue J , Brueckmann M , Eikelboom J , Themeles E , Ezekowitz M , Wallentin L , et al. Causes of death and influencing factors in patients with atrial fibrillation: a competing‐risk analysis from the randomized evaluation of long‐term anticoagulant therapy study. Circulation. 2013;128:2192–2201. doi: 10.1161/CIRCULATIONAHA.112.000491 24016454

[31] Pokorney SD , Piccini JP , Stevens SR , Patel MR , Pieper KS , Halperin JL , Breithardt G , Singer DE , Hankey GJ , Hacke W , et al. Cause of death and predictors of all‐cause mortality in anticoagulated patients with nonvalvular atrial fibrillation: data from ROCKET AF. J Am Heart Assoc. 2016;5:5. doi: 10.1161/JAHA.115.002197 PMC4943233 26955859

[32] Chen LY , Sotoodehnia N , Bůžková P , Lopez FL , Yee LM , Heckbert SR , Prineas R , Soliman EZ , Adabag S , Konety S , et al. Atrial fibrillation and the risk of sudden cardiac death: the Atherosclerosis Risk in Communities study and Cardiovascular Health Study. JAMA Intern Med. 2013;173:29–35. doi: 10.1001/2013.jamainternmed.744 23404043 PMC3578214

[33] Waldmann V , Jouven X , Narayanan K , Piot O , Chugh SS , Albert CM , Marijon E . Association between atrial fibrillation and sudden cardiac death. Circ Res. 2020;127:301–309. doi: 10.1161/CIRCRESAHA.120.316756 32833581

[34] Huikuri HV , Castellanos A , Myerburg RJ . Sudden death due to cardiac arrhythmias. N Engl J Med. 2001;345:1473–1482. doi: 10.1056/NEJMra000650 11794197

[35] Aro AL , Anttonen O , Tikkanen JT , Junttila MJ , Kerola T , Rissanen HA , Reunanen A , Huikuri HV . Prevalence and prognostic significance of T‐wave inversions in right precordial leads of a 12‐lead electrocardiogram in the middle‐aged subjects. Circulation. 2012;125:2572–2577. doi: 10.1161/CIRCULATIONAHA.112.098681 22576982

[36] Kim AHJ , Kim B , Rhee S , jin LY , Park JS , Lee SM , Kim SM , Lee SH , Yu KS , Jang IJ , et al. Assessment of induced CYP3A activity in pregnant women using 4β‐hydroxycholesterol: cholesterol ratio as an appropriate metabolic marker. Drug Metab Pharmacokinet. 2018;33:173–178. doi: 10.1016/j.dmpk.2018.04.004 29759884

[37] Haukilahti MAE , Holmstrom L , Vahatalo J , Kentta TV , Pakanen L , Tikkanen J , Perkiomaki JS , Rissanen H , Knekt P , Huikuri HV , et al. Association of isolated T inversion and sudden cardiac death – etiology and gender differences. Eur Heart J. 2020;41:ehaa946.2903. doi: 10.1093/EHJCI/EHAA946.2903

[38] Al‐Goblan AS , Al‐Alfi MA , Khan MZ . Mechanism linking diabetes mellitus and obesity. Diabetes Meta Syndr Obes. 2014;7:587. doi: 10.2147/DMSO.S67400 PMC4259868 25506234

[39] Kruse MS , Vega MC , Rey M , Coirini H . Sex differences in LXR expression in normal offspring and in rats born to diabetic dams. J Endocrinol. 2014;222:53–60. doi: 10.1530/JOE-14-0054 24824431

[40] DeLeon‐Pennell KY , Mouton AJ , Ero OK , Ma Y , Padmanabhan Iyer R , Flynn ER , Espinoza I , Musani SK , Vasan RS , Hall ME , et al. LXR/RXR signaling and neutrophil phenotype following myocardial infarction classify sex differences in remodeling. Basic Res Cardiol. 2018;113:40. doi: 10.1007/S00395-018-0699-5 30132266 PMC6105266

[41] Haukilahti MAE , Holmström L , Vähätalo J , Kentta T , Tikkanen J , Pakanen L , Kortelainen ML , Perkiömäki JS , Huikuri HV , Myerburg RJ , et al. Sudden cardiac death in women: causes of death, autopsy findings, and electrocardiographic risk markers. Circulation. 2019;139:1012–1021. doi: 10.1161/CIRCULATIONAHA.118.037702 30779638

