# Supplementary material for: Liver X Receptor Agonist 4β‐Hydroxycholesterol as a Prognostic Factor in Coronary Artery Disease
Source: J Am Heart Assoc. 2024 Feb 23;13(5):e031824. doi: 10.1161/JAHA.123.031824 (PMC10944077; doi:10.1161/JAHA.123.031824)
Supplement: Supplementary file 1 — Tables S1–S5 Figure S1 [file JAH3-13-e031824-s001.pdf]

Supplemental Material

Table S1. Cox regression analysis for 4β-hydroxycholesterol and sudden cardiac death with total cholesterol as a covariate.

**4βHC**	**Sudden Cardiac Death**							
	**Univariate**		**Model 1**		**Model 2**		**Model 3**	
	Hazard Ratio (95% CI)	p-value	Hazard Ratio (95% CI)	p-value	Hazard Ratio (95% CI)	p-value	Hazard Ratio (95% CI)	p-value
	**Males**							
Continuous (ln)	2.84 (1.53-5.25)	<0.001	1.51 (0.73-3.10)	0.271	1.56 (0.76-3.21)	0.226	1.58 (0.76-3.32)	0.220
1st quartile	1.35 (0.52-3.56)	0.538	1.89 (0.67-5.37)	0.228	1.87 (0.66-5.33)	0.239	2.20 (0.72-6.9)	0.165
2nd quartile	1.13 (0.41-3.12)	0.809	1.67 (0.56-4.97)	0.353	1.67 (0.56-4.99)	0.357	1.78 (0.55-5.8)	0.337
3rd quartile	*Reference*		*Reference*		*Reference*		*Reference*	
4th quartile	3.76 (1.62-8.72)	0.002	3.23 (1.25-8.33)	0.015	3.30 (1.27-8.53)	0.014	4.01 (1.43-11.1)	0.008
Cutoff >11.0 ng/ml	3.27 (1.85-5.75)	<0.001	2.29 (1.16-4.52)	0.017	2.34 (1.18-4.64)	0.015	2.65 (1.31-5.36)	0.006
	**Females**							
Continuous (ln)	0.41 (0.15-1.12)	0.083	0.40 (0.12-1.40)	0.154	0.44 (0.13-1.50)	0.188	0.44 (0.13-1.51)	0.192
1st quartile	4.51 (0.52-38.7)	0.170	7.29 (0.58-91.0)	0.123	8.03 (0.60-106)	0.114	13.1 (0.62-279)	0.099
2nd quartile	7.79 (0.97-62.4)	0.052	10.7 (1.02-112)	0.048	12.6 (1.13-142)	0.040	23.0 (1.23-440)	0.037
3rd quartile	1.97 (0.17-21.7)	0.579	3.26 (0.26-40.4)	0.356	3.56 (0.28-45.0)	0.326	4.78 (0.27-84)	0.284
4th quartile	*Reference*		*Reference*		*Reference*		*Reference*	
Cutoff < 9.7 ng/ml	5.57 (1.26-24.5)	0.023	5.99 (1.09-32.9)	0.040	7.20 (1.19-43.7)	0.032	10.7 (1.25-92.3)	0.030

Hazard ratios with 95% confidence intervals (CI) were calculated by univariate Cox regression analysis for 4βHC and sudden cardiac death. Cox regression where age, body mass index, type 2 diabetes, Canadian Cardiovascular Society grading of angina pectoris, left ventricular ejection fraction, total cholesterol, albumin-creatinine ratio, creatinine clearance, Hemoglobin A1C, high-sensitive C-reactive protein, highly sensitive troponin, soluble ST2, B-type natriuretic peptide, and leisure time physical activity were entered in the model as continuous variables when applicable (Model-1). Second multivariate analysis was performed as model 1 + use of antihypertensive medication (model 2) (beta-adrenergic blocking agent, angiotensin converting enzyme inhibitor, angiotensin receptor II blocker, calcium channel blocker and diuretics). Third multivariate analysis was performed as model 2 + QTc interval and presence of T wave inversions in all inferior leads (II, III, aVF)

Table S2. Cox regression analysis for 4β-hydroxycholesterol and sudden cardiac death without cholesterol as a covariate.

**4βHC**	**Sudden Cardiac Death**							
	**Univariate**		**Model 1**		**Model 2**		**Model 3**	
	Hazard Ratio (95% CI)	p-value	Hazard Ratio (95% CI)	p-value	Hazard Ratio (95% CI)	p-value	Hazard Ratio (95% CI)	p-value
	**Males**							
Continuous (ln)	2.84 (1.53-5.25)	<0.001	3.29 (1.74-6.21)	<0.001	3.33 (1.76-6.29)	<0.001	3.29 (1.71-6.31	<0.001
1st quartile	1.35 (0.52-3.56)	0.538	1.26 (0.46-3.40)	0.650	1.24 (0.46-3.36)	0.674	1.51 (0.52-4.38)	0.443
2nd quartile	1.13 (0.41-3.12)	0.809	1.16 (0.41-3.35)	0.774	1.15 (0.40-3.29)	0.798	1.26 (0.41-3.97)	0.328
3rd quartile	*Reference*		*Reference*		*Reference*		*Reference*	
4th quartile	3.76 (1.62-8.72)	0.002	4.66 (1.90-11.4)	<0.001	4.67 (1.89-11.5)	<0.001	5.45 (2.07-14.3)	<0.001
Cutoff >11.0 ng/ml	3.27 (1.85-5.75)	<0.001	4.21 (2.28-7.78)	<0.001	4.28 (2.30-7.96)	<0.001	4.59 (2.42-8.71)	<0.001
	**Females**							
Continuous (ln)	0.41 (0.15-1.12)	0.083	0.41 (0.13-1.28)	0.125	0.44 (0.14-1.33)	0.144	0.47 (0.15-1.44)	0.184
1st quartile	4.51 (0.52-38.7)	0.170	6.04 (0.59-62.3)	0.131	6.58 (0.59-74)	0.127	8.20 (0.51-132)	0.138
2nd quartile	7.79 (0.97-62.4)	0.052	10.7 (1.15-99)	0.037	12.2 (1.21-124)	0.034	16.3 (1.06-250)	0.045
3rd quartile	1.97 (0.17-21.7)	0.579	2.41 (0.20-29.5)	0.492	2.67 (0.21-34.0)	0.449	3.59 (0.20-63)	0.383
4th quartile	*Reference*		*Reference*		*Reference*		*Reference*	
Cutoff < 9.7 ng/ml	5.57 (1.26-24.5)	0.023	5.16 (1.05-25.4)	0.044	6.09 (1.13-33.0)	0.036	6.44 (1.04-40.1)	0.046

Hazard ratios with 95% confidence intervals (CI) were calculated by univariate Cox regression analysis for 4βHC and sudden cardiac death. Cox regression where age, body mass index, type 2 diabetes, Canadian Cardiovascular Society grading of angina pectoris, left ventricular ejection fraction, albumin-creatinine ratio, creatinine clearance, Hemoglobin A1C, high-sensitive C-reactive protein, highly sensitive troponin, soluble ST2, B-type natriuretic peptide, and leisure time physical activity were entered in the model as continuous variables when applicable (Model-1). Second multivariate analysis was performed as model 1 + use of antihypertensive medication (model 2) (beta-adrenergic blocking agent, angiotensin converting enzyme inhibitor, angiotensin receptor II blocker, calcium channel blocker and diuretics). Third multivariate analysis was performed as model 2 + QTc interval and presence of T wave inversions in all inferior leads (II, III, aVF)

Table S3. Characteristics of patients according to plasma 4α-hydroxycholesterol quartiles at baseline in men.

	**n=300**	**n=296**	**n=301**	**n=295**	**n=1192**
**4αHC (min-max)**	**0.9-3.4**	**3.4-4.4**	**4.4-5.7**	**5.7-14.5**	**p-value**
Age (years)	66 ± 8	66 ± 9	66 ± 9	66 ± 8	0.974
Body mass index (kg/m²)	27.5 ± 4.0	27.7 ± 4.0	28.4 ± 4.6	29.5 ± 4.4	<0.001
Resting systolic (mmHg))	142 ± 21	141 ± 23	145 ± 23	145 ± 22	0.088
Resting diastolic (mmHg)	80 ± 12	82 ± 10	81 ± 11	82 ± 12	0.054
Smokers (%)	29 (10)	26 (9)	29 (10)	26 (9)	0.969
Alcohol consumers (%)	121 (41)	122 (41)	136 (45)	138 (47)	0.336
Servings/week (if user)	5 (2-8)	5 (2-10)	4 (2-10)	4 (2-10)	0.960
History of AMI (%)	147 (49)	144 (49)	163 (54)	156 (53)	0.434
History of PCI/CABG (%)	248 (83)	241 (82)	251 (84)	247 (84)	0.876
Syntax Score	0 (0-5)	2 (0-6)	2 (0-7)	2 (0-5)	0.115
CCS class ≥ 2 (%)	91 (30)	95 (32)	124 (41)	118 (40)	0.008
Leisure time physical activity					<0.001
Highly active (%)	65 (22)	61 (21)	36 (12)	51 (17)	
Active (%)	124 (41)	109 (37)	115 (38)	85 (29)	
Irregularly active (%)	93 (31)	107 (36)	110 (37)	119 (40)	
Inactive (%)	18 (6)	19 (6)	40 (13)	40 (14)	
Relative METs	86±20	85±19	81±21	78±21	<0.001
Type 2 diabetes mellitus (%)	95 (32)	98 (33)	133 (44)	185 (63)	<0.001
Duration of diabetes (years)	4 (1-12)	5 (1-15)	6 (1-12)	6 (2-12)	0.876
**Echocardiogram parameters**					
Left ventricular ejection action (%)	64 ± 9	63 ± 10	63 ± 10	63 ± 10	0.651
Left ventricular mass (g)	216 ± 52	221 ± 60	226 ± 61	229 ± 60	0.041
Septal thickness at diastole (mm)	11.1 ± 1.8	11.4 ± 2.1	11.6 ± 2.0	11.8 ± 2.1	<0.001
Lateral wall thickness (mm)	10.3 ± 1.5	10.6 ± 1.7	10.7 ± 1.7	10.9 ± 1.7	<0.001
Diastolic function E/E’	9.9 ± 3.5	9.6 ± 3.5	10.0 ± 3.3	10.0 ± 3.7	0.120
**Laboratory analyses**					
Glycated hemoglobin (mmol/mol)	6.2 ± 1.0	6.2 ± 0.9	6.3 ± 0.9	6.7 ± 1.3	<0.001
Total cholesterol (mmol/L)	3.5 ± 0.6	3.7 ± 0.6	3.9 ± 0.8	4.0 ± 0.8	<0.001
High-density lipoprotein (mmol/L)	1.2 ± 0.3	1.2 ± 0.2	1.2 ± 0.3	1.2 ± 0.3	0.011
Low-density lipoprotein (mmol/L)	2.0 ± 0.5	2.1 ± 0.5	2.3 ± 0.7	2.3 ± 0.7	<0.001
Triglycerides (mmol/L)	1.0 (0.8-1.3)	1.1 (0.9-1.5)	1.2 (0.9-1.7)	1.5 (1.1-2.2)	<0.001
Creatinine clearance (mL/min)	96 ± 33	96 ± 33	99 ± 39	104 ± 36	0.019
U-albumin/creatinine-ratio	0.7 (0.5-1.1)	0.8 (0.5-1.1)	0.8 (0.5-1.2)	0.8 (0.5-1.6)	0.058
hs-CRP (mg/mL)	0.8 (0.4-1.9)	0.8 (0.5-1.5)	1.0 (05-2.1)	0.9 (0.5-2.1)	0.002
hs-TnT (ng/L)	8 (6-13)	9 (6-16)	10 (6-16)	9 (7-14)	0.007
BNP (ng/L)	42 (21-85)	47 (23-91)	46 (21-98)	43 (25-79)	0.587
sST2(ng/L)	17 (13-23)	16 (13-21)	17 (14-23)	19 (14-25)	<0.001
Galectin-3 (ng/L)	11 (9-13)	11 (9-13)	11 (9-13)	11 (9-13)	0.577
ALT (IU/L)	26 (20-34)	28 (22-37)	28 (21-36)	28 (21-39)	0.118
GGT (IU/L)	26 (15-40)	28 (20-40)	31 (22-47)	36 (21-61)	<0.001
**Medication**					
β-blockers (%)	262 (88)	260 (88)	264 (88)	270 (92)	0.337

ACE inhibitors or ATII blockers (%)	202 (67)	205 (69)	204 (61)	201 (68)	0.964
Calcium channel blockers (%)	66 (22)	67 (23)	69 (23)	87 (30)	0.114
Diuretics (%)	80 (27)	89 (30)	91 (30)	107 (36)	0.083
Psychotropic agents (%)	15 (5)	18 (6)	19 (6)	27 (9)	0.211
**Electrocardiogram parameters**					
Ventricular rate (bpm)	58 ± 9	58 ± 9	61 ± 10	62 ± 10	<0.001
PQ interval (ms)	184±32	186±35	183±31	184±34	0.615
QRS duration (ms)	107±18	104±15	106±18	105±18	0.356
QT interval (ms)	427±33	429±31	421±34	421±33	0.003
QTc interval (ms)	418±26	421±27	423±28	426±26	0.003
T wave inversion in leads (%)					
II	26 (8.9)	24 (8.3)	39 (13.3)	42 (14.5)	0.038
III	141 (48.3)	115 (39.8)	125 (42.7)	121 (41.7)	0.192
aVF	51 (17.5)	40 (13.8)	50 (17.1)	52 (17.9)	0.539
T wave inversion in all leads (%)					
II, III and aVF	16 (5.3)	15 (5.1)	25 (8.3)	28 (9.5)	0.088

Values are mean (SD), median (1st-3rd quartile) or n (% within group). 4αHC indicates 4α-hydroxycholesterol; AMI, Acute myocardial infarction; PCI/CABG, Primary coronary intervention/Coronary artery bypass grafting; CCS, Canadian Cardiovascular Society grading of angina pectoris; MET, Metabolic equivalents; hs-CRP, high-sensitivity C-reactive protein; hs-TnT, High-sensitivity cardiac troponin; BNP, B-type natriuretic peptide; sST2, Soluble ST2; ALT; Alanine aminotransferase; GGT, Gamma-glutamyl transferase; β-blocker, beta-adrenergic blocking agents; ACE inhibitor, angiotensin converting enzyme inhibitor; ATII blocker, angiotensin receptor II blocker.

Table S4. Characteristics of patients according to plasma 4α-hydroxycholesterol quartiles at baseline in women.

	**n=135**	**n=137**	**n=137**	**n=136**	**n=545**
**4αHC (min-max)**	**1.93-3.8**	**3.8-4.9**	**4.9-6.4**	**6.4-14.4**	**p-value**
Age (years)	68 ± 9	70 ± 8	70 ± 7	69 ± 7	0.053
Body mass index (kg/m²)	27.4 ± 5.0	28.1 ± 4.5	28.7 ± 5.0	29.5 ± 5.1	0.005
Resting systolic (mmHg))	154 ± 26	152 ± 27	157 ± 26	152 ± 27	0.398
Resting diastolic (mmHg)	80 ± 11	79 ± 12	80 ± 12	80 ± 12	0.544
Smokers (%)	8 (6)	5 (4)	5 (4)	12 (9)	0.195
Alcohol consumers (%)	25 (19)	22 (25)	16 (12)	26 (19)	0.331
Servings/week (if user)	2 (1-4)	1 (1-3)	2 (1-4)	2 (1-4)	0.786
History of AMI (%)	54 (40)	64 (47)	55 (40)	66 (49)	0.356
History of PCI/CABG (%)	105 (78)	100 (73)	110 (80)	109 (80)	0.430
Syntax Score	0 (0-2)	0 (0-5)	0 (0-4)	2 (0-6)	0.002
CCS class ≥ 2 (%)	72 (53)	75 (55)	79 (58)	84 (62)	0.512
Leisure time physical activity					0.486
Highly active (%)	16 (12)	22 (16)	16 (12)	15 (11)	
Active (%)	52 (39)	57 (42)	59 (43)	49 (36)	
Irregularly active (%)	52 (39)	47 (34)	52 (38)	51 (38)	
Inactive (%)	15 (11)	11 (8)	10 (7)	21 (15)	
Relative METs	88 (21)	90 (20)	88 (21)	82 (20)	0.010
Type 2 diabetes mellitus (%)	45 (33)	39 (29)	57 (42)	80 (60)	<0.001
Duration of diabetes (years)	8 (4-17)	3 (0-11)	7 (2-10)	6 (3-12)	0.021
**Echocardiogram parameters**					
Left ventricular ejection action (%)	66 ± 8	68 ± 7	66 ± 7	65 ± 10	0.026
Left ventricular mass index (g)	170 ± 44	166 ± 43	173 ± 42	183 ± 54	0.012
Septal thickness at diastole (mm)	10.2 ± 2.1	10.3 ± 2.1	10.5 ± 1.7	10.9 ± 2.3	0.022
Lateral wall thickness (mm)	9.5 ± 1.5	9.6 ± 1.7	9.7 ± 1.3	9.9 ± 1.8	0.120
Diastolic function E/E’	11.6 ± 4.3	11.6 ± 3.9	11.6 ± 4.1	12.7 ± 4.6	0.099
**Laboratory analyses**					
Glycated hemoglobin (mmol/mol)	6.2 ± 0.8	6.2 ± 0.8	6.3 ± 1.1	6.5 ± 0.9	0.003
Total cholesterol (mmol/L)	3.9 ± 0.6	4.1 ± 0.6	4.1 ± 0.7	4.3 ± 0.8	<0.001
High-density lipoprotein (mmol/L)	1.4 ± 0.3	1.4 ± 0.3	1.4 ± 0.4	1.4 ± 0.4	0.410
Low-density lipoprotein (mmol/L)	2.1 ± 0.6	2.2 ± 0.6	2.2 ± 0.6	2.4 ± 0.7	<0.001
Triglycerides (mmol/L)	1.1 (0.9-1.5)	1.2 (0.9-1.5)	1.1 (0.7-1.5)	1.5 (1.2-2.0)	<0.001
Creatinine clearance (mL/min)	83 ± 28	76 ± 26	84 ± 28	85 ± 31	0.042
U-albumin/creatinine-ratio	1.2 (0.8-1.7)	1.2 (0.8-1.8)	1.2 (0.7-1.5)	1.1 (0.8-1.6)	0.616
hs-CRP (mg/mL)	0.8 (0.4-1.3)	0.9 (0.5-1.8)	1.0 (0.5-2.0)	1.5 (0.7-3.4)	<0.001
hs-TnT (ng/L)	6 (5-11)	7 (5-12)	7 (5-10)	8 (5-12)	0.122
BNP (ng/L)	65 (31-108)	58 (33-96)	53 (31-100)	59 (34-98)	0.658
sST2(ng/L)	13 (10-18)	14 (11-20)	15 (12-20)	17 (13-21)	<0.001
Galectin-3 (ng/L)	12 (10-14)	13 (11-16)	12 (10-15)	13 (10-16)	0.075
ALT (IU/L)	21 (16-27)	22 (17-30)	23 (18-29)	25 (18-33)	0.015
GGT (IU/L)	20 (14-30)	22 (15-35)	24 (17-38)	26 (18-42)	<0.001
**Medication**					
β-blockers (%)	113 (84)	122 (89)	120 (88)	126 (93)	0.147
ACE inhibitors or ATII blockers (%)	93 (69)	88 (64)	102 (75)	101 (74)	0.195

Calcium channel blockers (%)	30 (22)	28 (20)	38 (28)	45 (33)	0.072
Diuretics (%)	50 (37)	53 (39)	59 (43)	71 (52)	0.053
**Electrocardiogram parameters**					
Ventricular rate (bpm)	60 ± 8	60 ± 8	61 ± 10	61 ± 10	0.284
PQ interval (ms)	175±34	175±26	174±28	176±32	0.954
QRS duration (ms)	99±15	101±19	98±15	99±17	0.555
QT interval (ms)	428±34	431±35	427±36	428±32	0.766
QTc interval (ms)	426±24	428±26	431±21	432±25	0.192
T wave inversion in leads (%)					
II	16 (12.1)	11 (8.1)	14 (10.4)	20 (15.3)	0.318
III	51 (38.6)	57 (42.2)	50 (37.3)	41 (32.1)	0.390
aVF	27 (20.5)	21 (15.6)	19 (14.2)	14 (10.7)	0.171
T wave inversion in all leads (%)					
II, III and aVF	12 (8.9)	9 (6.6)	9 (6.6)	10 (7.4)	0.870

Values are mean (SD), median (1st-3rd quartile) or n (% within group). 4αHC indicates 4α-hydroxycholesterol; AMI, Acute myocardial infarction; PCI/CABG, Primary coronary intervention/Coronary artery bypass grafting; CCS, Canadian Cardiovascular Society grading of angina pectoris; MET, Metabolic equivalents; hs-CRP, high-sensitivity C-reactive protein; hs-TnT, High-sensitivity cardiac troponin; BNP, B-type natriuretic peptide; sST2, Soluble ST2; ALT; Alanine aminotransferase; GGT, Gamma-glutamyl transferase; β-blocker, beta-adrenergic blocking agents; ACE inhibitor, angiotensin converting enzyme inhibitor; ATII blocker, angiotensin receptor II blocker.

Table S5. Baseline values for 4βHC and 4αHC with Pearson correlation coefficients.

	**4βHC**		**4αHC**	
**Men (n=1192)**				
Median (ng/ml)	8.02		4.42	
Min (ng/ml)	1.23		0.87	
Max (ng/ml)	35.7		14.5	
Interquartile range (ng/ml)	4.60		2.34	
**Women (n=546/545)**				
Median (ng/ml)	9.21		4.85	
Min (ng/ml)	1.45		1.93	
Max (ng/ml)	49.1		14.4	
Interquartile range (ng/ml)	6.13		2.60	
	**Correlations of 4βHC with 4αHC and cholesterol**			
	r	*P* value	r	*P* value
**Men, 4βHC**	**4αHC**		**Total cholesterol**	
	0.136	<0.001	0.470	<0.001
**Women, 4βHC**	0.067	0.117	0.412	<0.01

Median, min-max and interquartile range as well as the correlation coefficients between 4βHC, 4αHC and total cholesterol for men and women.

Figure S1. Flowchart representing ARTEMIS study population.

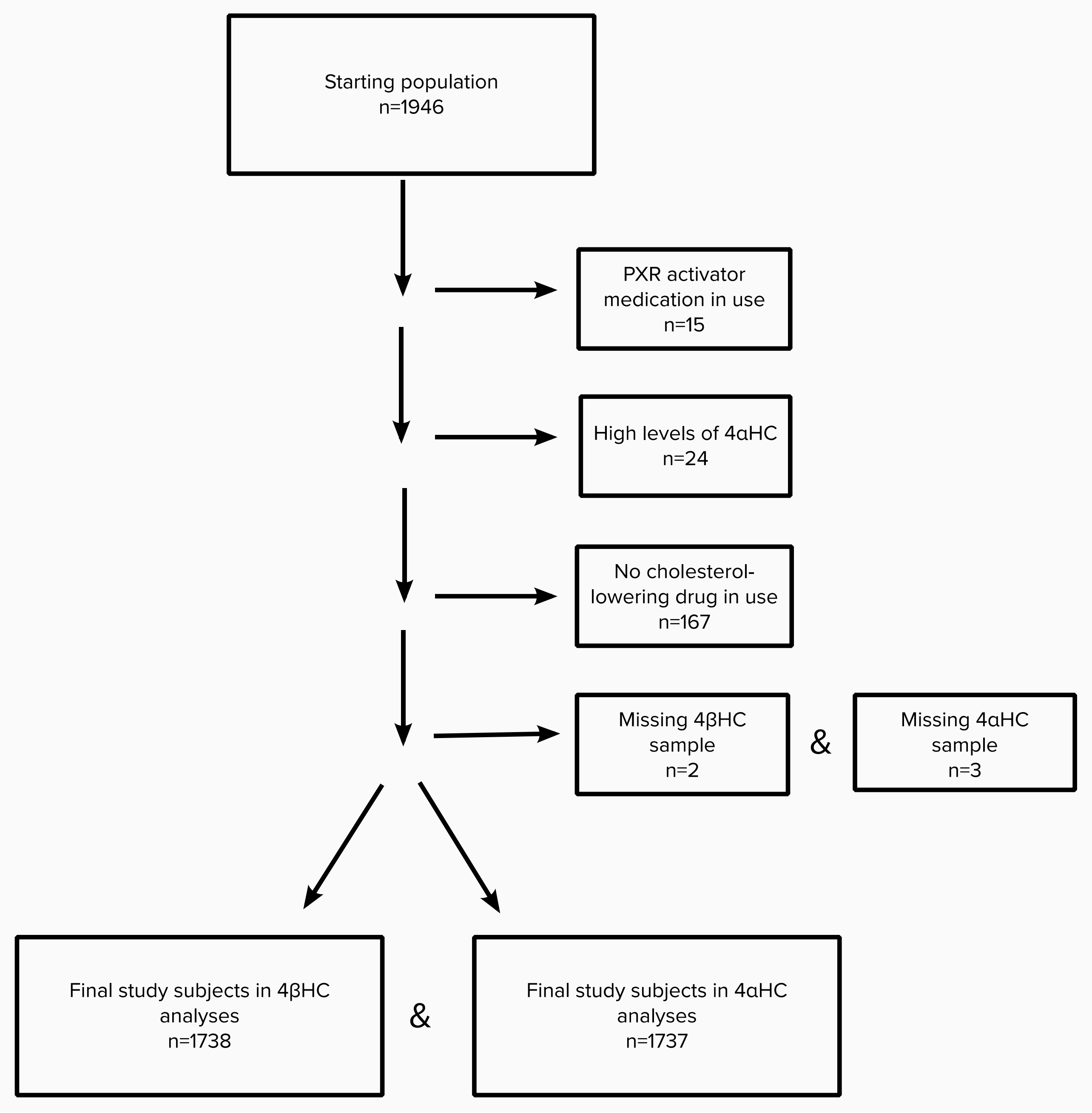

Of the initial total population (n=1946), 206 subjects were excluded. Excluded subpopulations were 1) Subjects with PXR activator medication, 2) Subjects with high levels of 4αHC as a marker of sample degradation, and 3) Subjects without cholesterol-lowering drug. Final number of subjects in analyses were n=1738 and n=1737, since two 4βHC samples and three 4αHC samples were missing. PXR indicates pregnane X receptor; 4βHC, 4β-hydroxycholesterol; 4α-hydroxycholesterol, 4αHC.
